# Delaying Health Care Due to the COVID-19 Pandemic: Associations With Physical and Mental Health and Preventive Care

**DOI:** 10.1093/geroni/igab046.1762

**Published:** 2021-12-17

**Authors:** Felicia Wheaton, Terika Scatliffe, Matilda Johnson

**Affiliations:** 1 Xavier University of Louisiana, New Orleans, Louisiana, United States; 2 Bethune-Cookman University, Daytona Beach, Florida, United States

## Abstract

Health care is important for maintaining optimal physical and mental health. However, due to the COVID-19 pandemic, many older adults have delayed or postponed care. Data from the special midterm release of the 2020 Health and Retirement Study (HRS) were used to examine the relationship between chronic conditions and delayed care, as well as between delayed care and mental health outcomes and preventative care among Americans aged 50+ (N=3,266). Approximately 30% of respondents said yes when asked “Since March 2020, was there any time when you needed medical or dental care, but delayed getting or did not get it at all?” Of those, 55% said their provider cancelled, closed or suggested rescheduling, 28.5% decided it could wait, and 20.8% were afraid to go. Results from OLS and logistic regression, controlling for sociodemographic characteristics, indicate that those with lung disease and those with a heart condition had significantly higher odds of delaying care. Delaying care was associated with significantly higher odds of poor self-rated health and feeling depressed, as well as significantly higher average hopelessness, loneliness and negative affect and significantly lower average positive affect. Surprisingly, delaying care was not associated with receiving a flu shot, cholesterol test, colonoscopy, mammogram or prostate exam in the previous two years. It is likely that the full effects of delaying health care during the pandemic have yet to be felt and there is a need to study the implications of such delays.

